# Multi-Irradiance: A Method for Simultaneous Measurement of the Temperature and Spectral Emissivity of High-Temperature Targets in SWIR

**DOI:** 10.3390/s22218469

**Published:** 2022-11-03

**Authors:** Yehan Sun, Jun Pan, Lijun Jiang, Libo Hao, Yu Cao, Helin Wang

**Affiliations:** College of Geo-Exploration Science & Technology, Jilin University, Changchun 130012, China

**Keywords:** high-temperature target, spectral emission, SWIR, multi-irradiance, emissivity–temperature separation

## Abstract

The currently used energy methods in spectral emissivity measurement are susceptible to the difference in temperature between the target and the reference blackbody. It is also limited by the state of the observation target and observation. This paper introduces the irradiance condition, while using the correlation between the information of emission energy and reflected energy of the high-temperature target. Based on the principle of radiative transmission and energy conservation, the relationship between the emissivity and bidirectional reflectance factor (BRF) was used to perform the retrieval of emissivity and temperature. An experimental device was designed, and graphite and rock were considered to verify the feasibility of the experimental scheme. The error of emissivity and temperature of both targets were, respectively, less than 5% and 0.5%, due to the Lambertian assumption, and the systematic errors had negligible impact on the retrieval. This verifies that the experimental observation method and scheme is reasonable.

## 1. Introduction

Forest fires, prairie fires and volcanic eruptions are typical high-temperature targets. A high-temperature target has a temperature significantly higher than that of adjacent ground objects. It usually refers to a target having a temperature greater than 500 K [1]. The recognition and temperature determination of a high-temperature target are crucial for several applications such as environmental monitoring and disaster warning [2,3].

According to Wien’s displacement law, high-temperature targets have a more significant response in short-wave infrared (SWIR, 1.3–3 μm) compared with the normal temperature ground objects [4,5]. Remote sensing images in SWIR often tend to have a higher spatial resolution than the thermal infrared bands. This is more conducive to the identification and temperature determination of small-area high-temperature targets [1,4,6]. Therefore, SWIR is a sensitive band for remote sensing monitoring of high-temperature targets. 

The emissivity of high-temperature target spectroscopy is one of the most important parameters when using SWIR for high-temperature target pixel identification, temperature determination and high-temperature area extraction. However, it is often approximated by the black body assumption or the emissivity of a normal temperature target [1,2]. An uncertainty exists in the value of the spectral emissivity of the object, due to the fact that it is affected by several factors such as the material type, target state, thickness, and temperature [7,8,9,10]. In addition, it is coupled with temperature [5,11,12]. Therefore, obtaining the spectrum emissivity in SWIR and performing the synchronous acquisition of temperature are scientific issues that should be solved in the field of observation.

Theoretically, the emissivity characteristics of objects can be deduced from Fresnel’s law and Kirchhoff’s law. In addition, an emissivity model of the thermal infrared direction of metal crystals and metal oxides can be developed [13,14]. The model physical parameters, such as the extinction coefficient and refractive index, are difficult to obtain in practice. Moreover, the emissivity of most non-crystal objects, such as rocks, cannot be easily derived by theory. Therefore, experimental observation is the best approach for verifying the emissivity derived from the theory. It is also the most efficient method when the emissivity of targets cannot be theoretically deduced.

In terms of experimental observation, the current spectral emissivity measurement of high-temperature targets is mainly based on energy or reflectance [15,16]. Emissivity can be calculated as the ratio of the target radiant energy to the radiant energy of the blackbody at the same temperature. Most of the blackbody radiation energy is obtained by observing a blackbody furnace at the same temperature. The integral blackbody technique [15] and independent blackbody [16,17,18] with Fourier-transform infrared spectrometer are the main current approaches used for observation in SWIR. The integrated blackbody formed by the sample surface on the cavity bottom is coupled with the cavity wall. The independent blackbody performs the target and blackbody isothermal observation by synchronously heating the blackbody and the sample. This method is more suitable for material measurement on flat surfaces, and it inevitably has a high cost.

However, it is difficult to use blackbody furnaces to observe targets such as charcoal, flames, and magma, that are flammable and require on-site observation. Therefore, using the thermocouple temperature measurement method to obtain the blackbody radiance at the same temperature has become a better simplified alternative to the blackbody furnace in SWIR [7,9,19,20]. Similarly, temperature measurement using a thermocouple inevitably brings measurement errors [21,22].

According to Kirchhoff’s law, the indirect measurement of the target spectral emissivity can be performed by measuring the spectral reflectance. This method can avoid the errors caused by temperature measurement. Most of the existing studies observe the materials at normal or low temperatures using integrating spheres [23,24,25]. The radiation of the high-temperature target can easily interfere with the reflected light. In addition, the enclosed space is more suitable for material-scale sample observation. Moreover, some restrictions on the target objects that can be observed by remote sensing still exist, such as burning charcoal and molten rock.

This paper aims at synchronously retrieving the spectral emissivity and temperature, while avoiding the influence of the previously mentioned conditions, such as the temperature measurement and target state, on the observation of spectral emissivity. The irradiance condition was first introduced. The correlation between the reflected energy information and the target emission energy information (i.e., the correlation between the emissivity and the BRF) was then used. Based on the theory of radiative transfer and energy conservation, spectral emissivity and temperature inversion were performed. 

The remainder of this paper is organized as follows. Section 2 introduces the principle of spectral emissivity measurement using the multi-irradiance method. The design of the high-temperature target spectral emissivity observation platform and the experimental implementation results are detailed in Section 3 and Section 4. The experimental results and the error effects are discussed and analyzed in Section 5. Finally, the conclusions are drawn in Section 6.

## 2. Principle of the Multi-Irradiance Method for Spectral Emissivity Measurement

It is almost impossible to separate emissivity and temperature using a single equation. Therefore, a certain irradiance condition is introduced while using the resulting correlation between the reflected energy of the high-temperature target and the transmitted energy information, that is, the correlation between emissivity and the BRF. On the premise of not increasing the number of unknowns, the number of equations was increased in order to find the emissivity and temperature solution.

### 2.1. Spectral Emissivity

The spectral emissivity is the ratio of the target radiance L(λ,T) to the radiance of the blackbody $L_{b}\left(\lambda ,T\right)$ at the same temperature T:

(1) $$\varepsilon \left(\lambda ,T\right)=\frac{L\left(\lambda ,T\right)}{L_{b}\left(\lambda ,T\right)}$$

where  ε(λ,T) is the spectral emissivity of the target and λ represents the wavelength. Equation (1) can also be applied to the definition of directional spectral emissivity, that is, the ratio of target radiance to blackbody radiance at a certain observation angle. According to the Planck function, the directional emissivity can be expressed as:

(2) $$\varepsilon \left(\theta ,\varphi ;\lambda ,T\right)=\frac{L\left(\theta ,\varphi ;\lambda ,T\right)\lambda^{5}\left[\exp\left(\frac{hc}{\lambda kT}\right)-1\right]}{2hc^{2}}$$

where L(θ,φ;λ,T) is the directional radiance of the target at wavelength λ (in W m^−2^ ster^−1^ nm^−1^), T is the temperature (in K), h represents the Planck constant (h = 6.626 × 10^−34^ Js), c denotes the speed of light in a vacuum (c = 3 × 10^8^ m/s), and k is the Boltzmann constant (k = 1.381 × 10^−23^ J/K).

According to the principle of energy conservation, for an opaque object, the sum of the directional absorptance and the directional-hemispherical reflectance (DHR) is equal to 1. DHR refers to the special case of illumination from a collimated beam. It is the ratio of flux $d\Phi_{r}\left(\theta_{i},\varphi_{i}\right)$ reflected by a surface area dS from the target into a hemispherical to flux $d\Phi_{i}\left(\theta_{i},\varphi_{i}\right)$ of illumination [26]. It can be expressed as:

(3) $$DHR\left(\theta_{i},\varphi_{i}\right)=\frac{d\Phi_{r}\left(\theta ,\varphi \right)}{d\Phi_{i}\left(\theta ,\varphi \right)}$$

Moreover, *DHR* can be computed as [26]:

(4) $$DHR\left(\theta_{i},\varphi_{i}\right)=\int_{2\pi }^{}f_{r}\left(\theta ,\varphi ;\theta_{r},\varphi_{r}\right)cos\theta_{r}sin\theta_{r}d\theta_{r}d\varphi_{r}$$

where $\theta_{i},\varphi_{i};\theta_{r},\varphi_{r}$ are the zenith and azimuth angles of the direction of illumination and reflection and $f_{r}\left(\theta_{i},\varphi_{i};\theta_{r},\varphi_{r}\right)$ is the bidirectional reflectance distribution function (BRDF).

According to Kirchhoff’s law, if the system is in thermal equilibrium, then:

(5) $$\begin{array}{c} \varepsilon \left(\theta_{i},\varphi_{i};\lambda ,T\right)=\alpha \left(\theta_{i},\varphi_{i};\lambda ,T\right)\\ =1-DHR\left(\theta_{i},\varphi_{i};\lambda ,T\right)\\ =1-\int_{2\pi }^{}f_{r}\left(\theta_{i},\varphi_{i};\theta_{r},\varphi_{r}\right)cos\theta_{r}sin\theta_{r}d\theta_{r}d\varphi_{r} \end{array}$$

where $\alpha \left(\theta_{i},\varphi_{i};\lambda ,T\right)$ is the directional absorptance. 

### 2.2. Radiation Energy Equation of Multi-Irradiance Conditions

**Figure 1:**
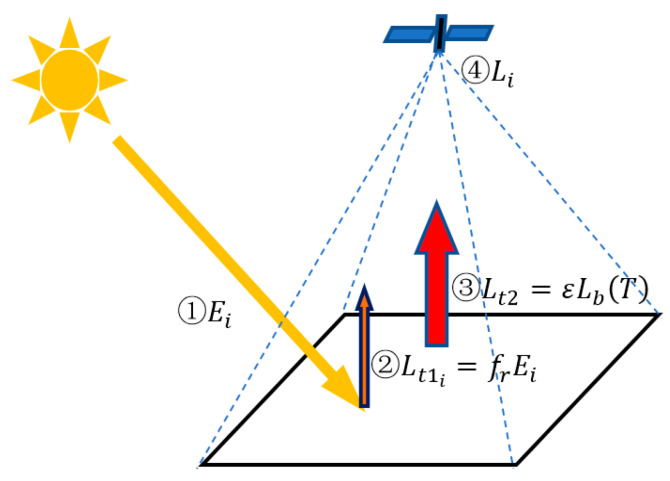
Illustration of the energy composition for multiple irradiance conditions. ① represents the incident energy of the light source, ② represents the reflected energy of the high-temperature target, ③ represents the emitted energy of the high-temperature target, and ④ represents the mixed energy received by the detector.

When there is no external lighting condition, the radiance L received by the detector is the emitted radiance $L_{t2}$ of the target (Figure 1). This means that two unknown quantities of emissivity and temperature exist in a single equation, which cannot be solved. Therefore, the irradiance condition is introduced, and the number of equations is increased in order to solve the problem of underdetermined equations. In other words, the radiance L, received by the detector in the direction of the zenith angle $\theta_{r}$ and azimuth angle $\varphi_{r},$ is a combination of the target reflected radiance $L_{t1}$ and emitted radiance of $L_{t2}$. Therefore, the radiant energy equation under multiple irradiance conditions is given by:

(6) $$L_{i}\left(\theta_{i},\varphi_{i};\theta_{r},\varphi_{r},T,\lambda \right)=L_{t1_{i}}\left(\theta_{i},\varphi_{i};\theta_{r},\varphi_{r},T,\lambda \right)+L_{t2}\left(\theta_{r},\varphi_{r},T,\lambda \right)\left(i=0,1,\ldots ,n\right)$$

where:

(7) $$L_{t1_{i}}\left(\theta_{i},\varphi_{i};\theta_{r},\varphi_{r},T,\lambda \right)=E_{i}\left(\theta_{i},\varphi_{i},\lambda \right)f_{r}\left(\theta_{i},\varphi_{i};\theta_{r},\varphi_{r},\lambda \right)$$

and:

(8) $$L_{t2}\left(\theta_{r},\varphi_{r},T,\lambda \right)=\varepsilon \left(\theta_{r},\varphi_{r},\lambda \right)L_{b}\left(\lambda \right)$$

where the light source is incident from the direction of the incident zenith angle $\theta_{i}$ and azimuth angle $\varphi_{i}$, the light source intensity is set to n levels, and $E_{i}\left(\lambda \right)$ is the irradiance from the illumination when its intensity level is *i*.

The BRDF is a non-measurable theoretical concept [26]. Therefore, the Lambertian surface (i.e., standard diffuse panel) is usually used to measure the BRF and indirectly measure the BRDF [26]:

(9) $$R\left(\theta_{i},\varphi_{i};\theta_{r},\varphi_{r},\lambda \right)=\pi f_{r}\left(\theta_{i},\varphi_{i};\theta_{r},\varphi_{r},\lambda \right)$$

and:

(10) $$E_{i}\left(\lambda \right)=\frac{d\Phi_{r}{}_{i}}{dS}=\frac{d\Phi_{i}{}_{i}}{dS}=\frac{\pi L_{e}{}_{i}\left(\theta_{i},\varphi_{i};\theta_{r},\varphi_{r}\right)dS}{dS}=\pi L_{e}{}_{i}\left(\theta_{i},\varphi_{i};\theta_{r},\varphi_{r}\right)\;$$

where $L_{e}{}_{i}\left(\theta_{i},\varphi_{i};\theta_{r},\varphi_{r}\right)$ is the reflected radiance of the standard diffuse panel in the same direction. 

Therefore, Equation (7) can be written as:

(11) $$L_{t1_{i}}\left(\theta_{i},\varphi_{i};\theta_{r},\varphi_{r},T,\lambda \right)=R\left(\theta_{i},\varphi_{i};\theta_{r},\varphi_{r},\lambda \right)L_{e}{}_{i}\left(\theta_{r},\varphi_{r}\right)$$

where Equations (9) and (10) are used.

When the target is the Lambertian surface, the radiance is the same in all directions, and the emissivity can be expressed as:

(12) $$\begin{array}{ll} \varepsilon \left(\theta_{r},\varphi_{r},\lambda ,T\right)= & 1-\int_{2\pi }^{}\frac{R\left(\theta_{i},\varphi_{i};\theta_{r},\varphi_{r}\right)}{\pi }cos\theta_{r}sin\theta_{r}d\theta_{r}d\varphi_{r}\\ & =1-R\left(\theta_{i},\varphi_{i};\theta_{r},\varphi_{r}\right) \end{array}$$

using Equations (5) and (9).

Therefore, for the Lambertian target, Equation (6) can be expressed as:

(13) $$\{\begin{array}{c} L_{0}\left(\lambda ,T\right)=\varepsilon \left(\lambda ,T\right)L_{b}\left(\lambda ,T\right)\\ L_{1}\left(\lambda ,T\right)=L_{e}{}_{1}\left(\lambda ,T\right)\left(1-\varepsilon \left(\lambda ,T\right)\right)+\varepsilon \left(\lambda ,T\right)L_{b}\left(\lambda ,T\right)\\ \ldots \ldots \\ L_{n}\left(\lambda ,T\right)=L_{e}{}_{n}\left(\lambda ,T\right)\left(1-\varepsilon \left(\lambda ,T\right)\right)+\varepsilon \left(\lambda ,T\right)L_{b}\left(\lambda ,T\right) \end{array}$$

Moreover,

(14) $$\varepsilon \left(\lambda ,T\right)=\frac{1}{n}\sum_{i=1}^{n}(\left(L_{i}\left(\lambda ,T\right)-L_{0}\left(\lambda ,T\right)\right)/L_{e}{}_{i}\left(\lambda ,T\right))$$

(15) $$T_{\lambda }=\frac{hc}{k\lambda }\ln{\left(\frac{2hc^{2}\varepsilon \left(\lambda ,T\right)}{\lambda_{j}^{5}L_{0}\left(\lambda ,T\right)}+1\right)}^{-1}$$

**Figure 2:**
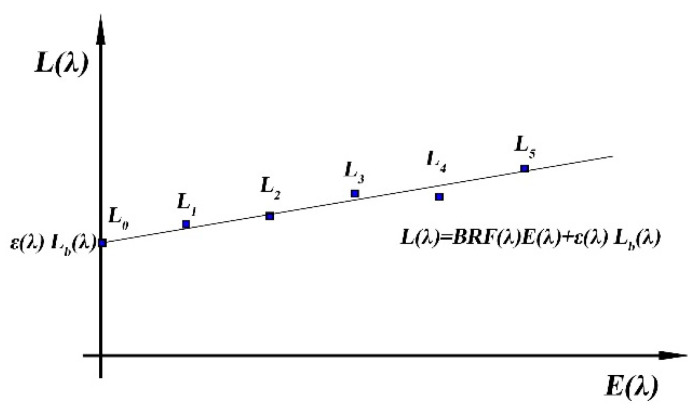
Geometric illustration of the multi-irradiance conditional energy equation system.

The quantitative relationship between the Lambertian BRF and emissivity is known, and only two unknown variables exist in the equations. Therefore, according to the two irradiance conditions (two sets of equations), the emissivity is first calculated (cf. Equation (14)), and the temperature is then retrieved using the Planck function (cf. Equation (15)). In the case where more than two conditions of irradiance exist, the least-squares fitting method can be used to eliminate the observation error of different irradiance conditions, and therefore obtain more accurate emissivity and temperature, as shown in Figure 2, which presents the geometric illustration of the multi-irradiance conditional energy equation system. The straight line represents the fitting process of all the observation points using the least-squares method. The intercept of the straight line is the radiance emitted by the high-temperature target, while the slope is the BRF of this band. 

## 3. Experimental Scheme Design Based on the Multi-Irradiance Method

The multi-irradiance method for emissivity observation was developed by introducing multi-irradiance conditions. According to the experimental principle, two or more irradiance experimental conditions were first built. The observation platform and experimental process were then designed, so as to perform the emission of high-temperature targets and acquire reflected energy information. Afterwards, the correlation between emissivity and BRF was used. Finally, the simultaneous retrieval of multi-irradiance emissivity-temperature was performed.

### 3.1. Instruments

The radiance of high-temperature targets was measured using the Hi-Res Pro portable ground spectrometer (FieldSpec4, Analytical Spectral Devices (ASD)., Inc., Longmont, CO, USA), with a spectral range of 350 nm–2500 nm. The spectral resolution was 3 nm in VNIR and 6 nm in SWIR. It was factory calibrated, and the spectral sampling interval was 1.4 nm in VNIR and 1.1 nm in SWIR with an error of 0.02% and 0.01%, respectively. The field of view of the spectrometer probe was 25°. It can be equipped with a lens converter in order to adjust the field of view. A 50 W halogen lamp was used as a light source.

The electric heating plate was used as a heating source in order to ensure that the target temperature was constant. The panel of the electric heating plate has a size of 10 cm × 10 cm. The electric heating source uses a proportional–integral–derivative controller (PID) intelligent temperature control system. The temperature can be set up to 1000 K. 

The surface temperature of the target was measured using a handheld digital thermometer (HH806AU, OMEGA Engineering Inc., Norwalk, CT, USA), cooperating with a handheld K-type thermocouple (88107K, OMEGA Engineering Inc.). The measurement temperature range is 0–760 °C, and the thermometer accuracy is 0.1% t ± 1 °C, where t is the measurement reading.

### 3.2. Experimental Device Design

The experiment should first ensure that there is no incident of external energy and stray light reflection influence. Therefore, the darkroom conditions were designed. The dark room wall covered the black body cloth whose reflectance is less than 0.02. The bottom of the darkroom should be laid with a high-temperature insulation layer of aluminum silicate ceramic fiber, in order to ensure safety. The slides were laid on the ground in order to facilitate the alternate measurement of the high-temperature targets and standard diffuse panel.

The multi-irradiance condition was then generated by the halogen light source with adjustable brightness. The incident angle of the light source can be adjusted according to the observation needs. Moreover, the light source intensity was attenuated with distance. It is necessary to ensure that the irradiance change caused by the light source would be greater than 10 times the instrument observation noise. Consequently, the noise would not cause measurement errors. Therefore, it was necessary to determine the appropriate light source power and the distance between the light source and the target to ensure the observation SNR.

**Figure 3:**
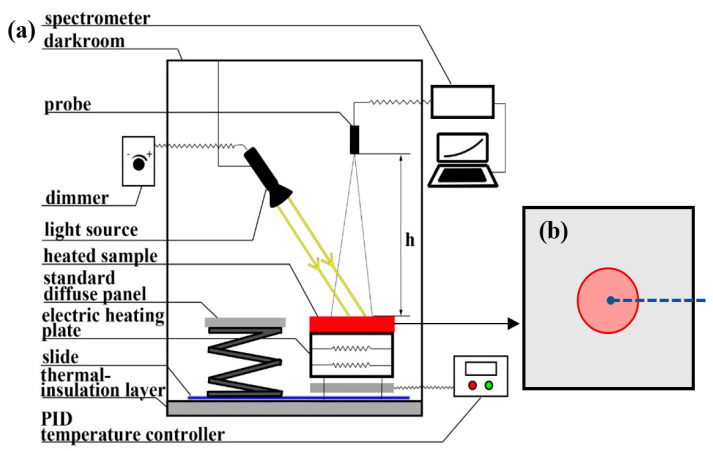
Experimental device design for (**a**,**b**) represents the surface of the observation target, in which the red area is the observation field of the spectrometer probe, the blue line represents the thermocouple and the blue point represents the temperature measurement point of the thermocouple.

Finally, the darkroom, light and observation conditions were combined (Figure 3a). The target radiance was observed by the ASD spectrometer, while the spectrometer observation probe was placed directly above the target. It is necessary to ensure that the observation target was fully in the observation field. Therefore, the height setting depends on the target size and the observation probe field-of-view angle. As long as the spectroscope is satisfied, the measurement will not be affected by the height. Note that the electric heating device was placed on the heat insulation layer, the observation target was placed on the electric heating plate, and the PID controller was placed outside the dark in order to prevent the stray light generated by the instrument from entering the observation field of view. In order to verify the sample emissivity and temperature inverted by the multi-irradiance method, the observed sample was laterally drilled and the thermocouple implanted into the hole to measure the target temperature, which is located at the center of the observation field of view (Figure 3b).

### 3.3. Experimental Procedure Design

The height and field of view of the spectrometer probe were first determined. The center of the spectral observation field can be located by the laser. Two lasers were placed on either side of the probe. The center of the line between two laser points was the center of the probe filed. The field radius was determined by the field of view and height. Once the observation field of the spectrometer is fixed, it is necessary to ensure that the relative position of the target and the spectrometer probe does not change, so as to avoid observation errors caused by different irradiance conditions that are caused by position conversion. For the observation of $L_{e}{}_{i}$, it was necessary to place the standard diffuse panel at the same height and position of the spectral observation field using the slide. The observation surfaces of the target can also be determined by the laser. $L_{e}{}_{i}$ can be obtained by dividing the observed radiance by the absolute reflectance of the standard diffuse panel. After completing the preparations, including the preheating of the instrument and heating of the high-temperature target to a stable state, the experiment process was designed as follows: 

(1) A high-temperature target spectral radiance observation ($L_{0}$) under zero irradiance conditions is considered; (2) the brightness of the light source is changed, the irradiance caused by the light source at the observation position of the high-temperature target is measured ($L_{e}{}_{i}$), and the mixed spectral radiance of the high-temperature target is observed ($L_{i}$); (3) the brightness of the light source is adjusted and step (2) is repeated in order to complete the observation of all the irradiance conditions; (4) after completing the spectrum measurement, the thermocouple temperature measurement is performed. 

## 4. Experimental Implementation and Data Collection

### 4.1. Experimental Samples

**Figure 4:**
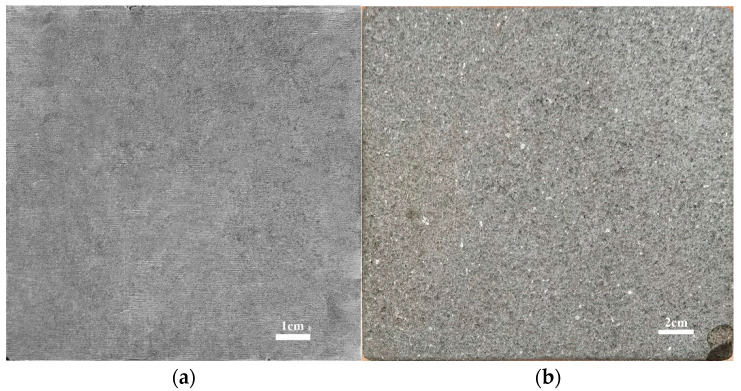
Samples of (**a**) graphite and (**b**) rock.

High-temperature graphite and high-temperature rocks, that were slightly directional and slightly Lambertian, were considered as observation targets for experimental observation and verification [27]. The graphite plate used was a high-purity graphite produced by Sanjing Carbon Co., Ltd. It has a length, width and height of 10 cm, 10 cm and 1 cm, respectively (Figure 4a). Its thermal conductivity, resistivity and density at normal temperature are 120 W/(m·K), 11 μΩ·m and 1.75 g/cm^3^, respectively. The surface of graphite is flat and uniform, with a steel gray color and metallic opaque luster, and it has extremely parallel cleavage. The measured roughness with Ra, Rq, and Rz are 6.92 ± 0.85 μm, 8.58 ± 0.84 μm, and 36.08 ± 2.83 μm, respectively.

The rock used was basaltic andesite from the Tengchong volcanic area of China, with a porphyritic and massive structure (Figure 4b). The phenocrysts mainly consist of 40% of plagioclase, 20% of pyroxene and 5% of amphibole, with pores developed and filled with calcite and zeolite. The surface is flat, with a length, width and height of 20 cm, 20 cm and 2 cm, respectively. The measured roughness with Ra, Rq, and Rz are 7.80 ± 1.00 μm, 9.93 ± 1.13 μm and 39.28 ± 6.37 μm, respectively.

### 4.2. Experimental Implementation

The zero irradiance and high irradiance conditions were selected for the observation of the normal spectral radiance of the high-temperature target in order to ensure that the observation had the highest signal-to-noise ratio and the minimum target temperature change. The light source was set at a solar altitude angle of 70° in order to simulate the summer sun conditions in the northern hemisphere, while the light spot was distributed on the high-temperature target. The field of view was set to 3°. The height of the spectrometer probe is 20 cm with a normal direction, and the observation field has a radius of 1.35 cm. The heated temperature was set to 773 k using a PID-controller. The target was heated by the electric heating plate. In order to ensure full heating of the target, it was necessary to continue the heating process for more than 10 min after the heating source became stable according to the experimental experience.

**Figure 5:**
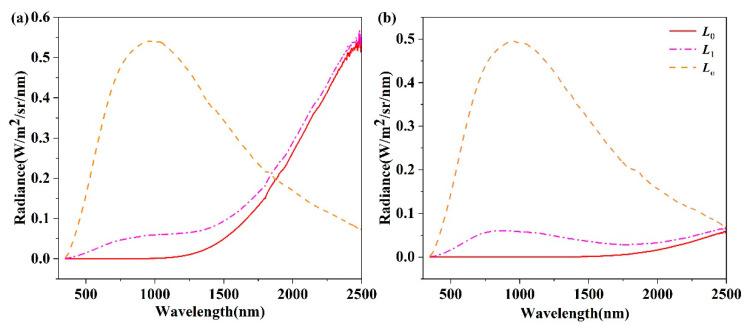
Radiance curve for (**a**) graphite and (**b**) rock. The yellow dot line represents $L_{e}$ , the pink dot-dash line represents $L_{1}$, and the red dash line represents $L_{0}$.

The center of each target was observed in 90 groups. In addition, each group included three parts of the observations of the target radiance with zero irradiance and high irradiance, as well as the radiance of the standard diffuse panel reflected light source. Moreover, 10 spectra were measured for each observation. These radiance data and temperature measurement data were then averaged. Figure 5 shows the target emitted radiance, the emitted and reflected mixed radiance of the target, and the radiance from the light source reflected by the standard diffuse panel. It can be seen that the observed target radiance is significantly different when illumination exists. 

According to Equations (14) and (15), the simultaneous retrieval temperature and spectral emissivity was performed. Since each band can retrieve a target temperature value from the spectral emissivity, the retrieved temperatures from each band of SWIR were averaged and then considered as the retrieved temperature of the target:

(16) $$\varepsilon \left(\lambda ,T\right)=\left(L_{1}\left(\lambda ,T\right)-L_{0}\left(\lambda ,T\right)\right)/L_{e}\left(\lambda ,T\right)$$

(17) $$\bar{T}=\frac{1}{2201}\sum_{\lambda =1300}^{2500}T_{\lambda }=\frac{1}{2201}\sum_{\lambda =1300}^{2500}\frac{hc}{k\lambda }\ln{\left(\frac{2hc^{2}\varepsilon \left(\lambda ,T\right)}{\lambda_{j}^{5}L_{0}\left(\lambda ,T\right)}+1\right)}^{-1}$$

where $L_{0}\left(\lambda ,T\right)$ and $L_{1}\left(\lambda ,T\right)$ are, respectively, the radiance of the target with zero irradiance and high irradiance, $L_{e}\left(\lambda ,T\right)$ is the radiance of the standard diffuse panel reflected light source, and $\bar{T}$ is the retrieved temperature of the target.

## 5. Results and Discussions

The experimental multi-irradiance method device was designed and used to perform the simultaneous inversion of emissivity and temperature for graphite and rock materials. The precision and error analysis of the retrieved spectrum emissivity and temperature were performed in order to verify the feasibility of the scheme.

### 5.1. Results of Spectral Emissivity–Temperature Synchronization Retrieval

**Figure 6:**
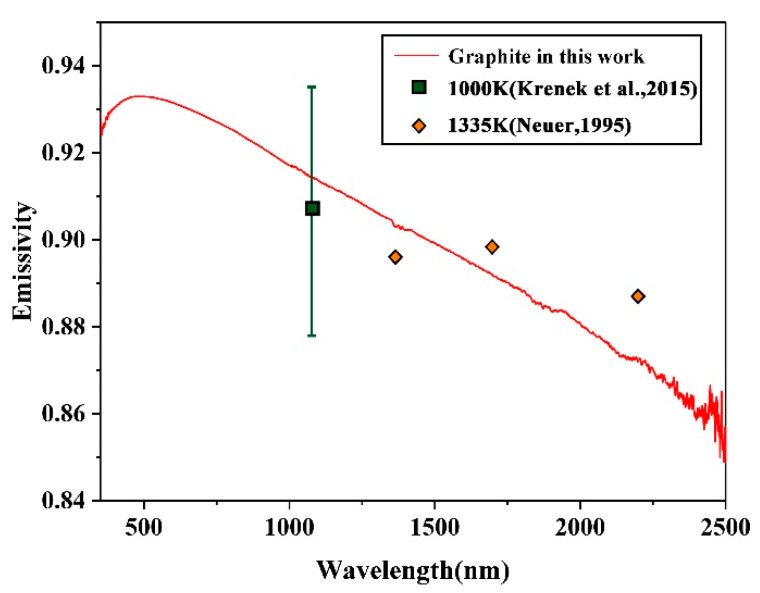
Graphite spectral emissivity. The red solid line represents the spectral emissivity of high-temperature graphite measured in this study. The yellow point represents the emissivity of 1335 K graphite observed by Neuer [10], and the green point represents the 1000 K graphite emissivity at 1064 nm observed by Krenek et al. [28].

**Figure 7:**
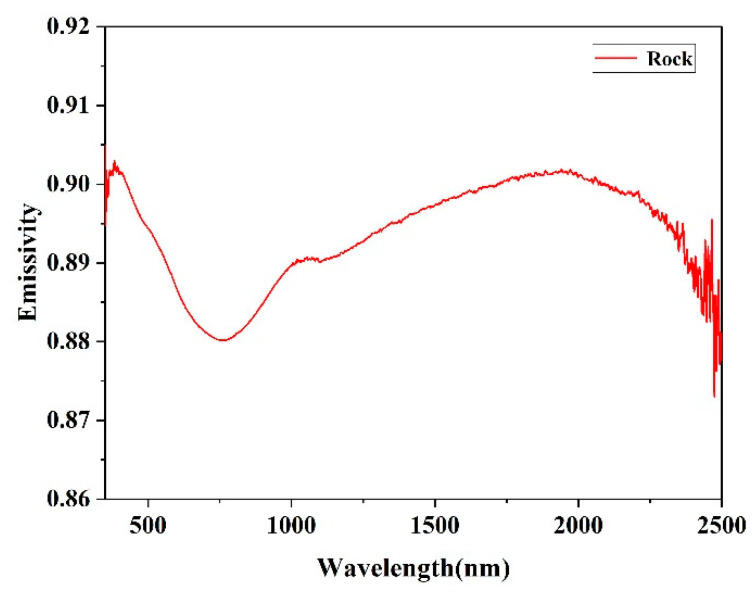
Rock spectral emissivity.

The normal spectral emissivity of graphite is mainly distributed in the visible-near infrared band (350–2500 nm) between 0.86 and 0.93. In addition, the emissivity slowly decreases with the increase of the wavelength, while slight fluctuations exist near the 1400 nm and 1900 nm bands (Figure 6). The normal emissivity of the rock is mainly distributed between 0.88 and 0.91, and the emissivity slightly varies with the wavelength. A tiny emission valley exists near the 750 nm band. When the wavelength increases, the emissivity slowly recovers to almost 0.9 as the wavelength increases (Figure 7).

The rationality of the emissivity experiment results can be demonstrated in the relevant literature, where the rationality of the experimental scheme is verified. The measurements of graphite can be compared to those of Neuer [10] and Krenek et al. [28], who investigated graphite material (Figure 6). The measurements of rock can be compared to those of Li et al. [8], who investigated rocks sampled from magmatic rocks in Antarctica, at 760 K and 875 K. Although there are differences in the mineral composition of these samples, their lithology is similar. The spectral emissivity detected by the two sets of experiments both exhibit stationarity in SWIR, and the average relative difference does not exceed 7% [8] (Figure 7). It can be seen that the measurements are generally coherent. 

**Figure 8:**
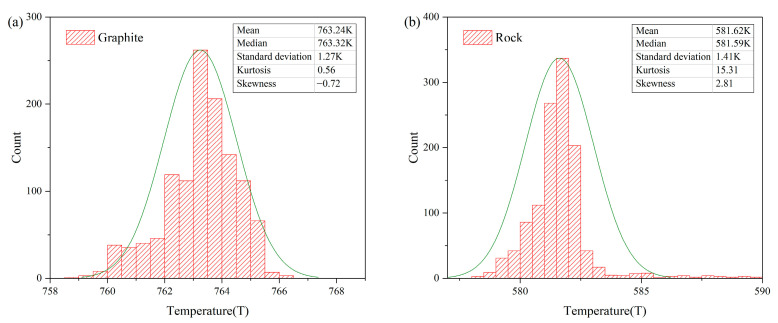
Temperature histogram retrieved in SWIR frequency distribution for graphite (**a**) and rock (**b**).

A temperature was retrieved for each wavelength based on Equation (16). The two sets of temperatures of the graphite and the rock, retrieved from each of the 1201 bands of SWIR (1300–2500 nm), have mean values with standard deviations of 763.24 ± 1.21 K and 581.62 ± 1.41 K, and the corresponding coefficients of variations (CVs), representing the ratio of standard deviation to mean, were equal to 0.17% and 0.24%, respectively (Figure 8). The mean value was also defined as the temperature retrieved of the targets. This reveals a small difference of retrieved temperature in each band. It can be observed from Table 1 that the retrieved temperature was slightly lower (by 2–3 K) than the thermocouple measurement temperature (Table 1). When the inner temperature is measured using thermocouples, it is usually higher than the surface temperature of the target due to the surface heat exchange [21]. Therefore, the thermocouple temperature is used as a reference for the approximate range of the target temperature. In addition, the retrieved temperature was lower than the thermocouple measurement temperature, which also shows the rationality of the temperature inversion results.

### 5.2. Error Analysis of Emissivity–Temperature Retrieval

If the observation conditions are in full compliance with the ideal assumptions, the retrieved temperatures by the observed spectral emissivity in each band should have the same value. However, not only the inversion temperature of a single band fluctuates, but also the inversion temperature between each band practically fluctuates to a certain extent. These fluctuations of temperatures retrieved from all the SWIR bands, that can also be considered as errors, originate from systematic errors, as well as errors in emissivity estimation due to the Lambertian assumption. Therefore, the error analysis was mainly conducted from these two aspects.

#### 5.2.1. Analysis of Systematic Errors

Some uncertainties exist in the temperature inversion results of each band. They are mainly caused by systematic errors, including the instrument errors, and the errors caused by the external environment and human operations of the experimenter during the observation process. When the emissivity error is ignored, according to Equation (15), the temperature uncertainty $u_{T}\left(\lambda \right)$ can be computed as [29]:

(18) $$u_{T}\left(\lambda \right)=\frac{dT\left(\lambda \right)}{dL_{0}\left(\lambda \right)}u_{L_{0}}$$

where $u_{L_{0}}$ is the variation of radiance caused by 0.01% error of the instrument instrumental error of 0.01% and $u_{L_{0}}=0.0001L_{0}$. 

**Figure 9:**
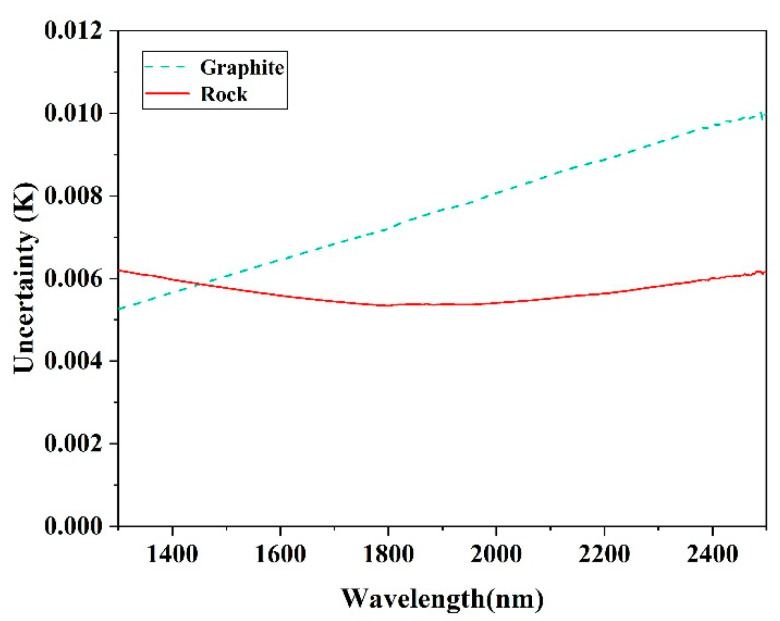
Temperature uncertainty in SWIR. The green dotted line and red solid line represent the uncertainty of graphite and rock, respectively.

Figure 9 shows that the temperature uncertainties of graphite and rock in SWIR were less than 0.01 K, which demonstrates the subtle effect of the systematic error from each band on retrieval.

The dispersion degree of the temperatures retrieved from each of the SWIR bands can reflect the influence of the systematic errors. The influence can be calculated by simulation when the errors of radiance ($L_{0}$) are introduced. Therefore, the mean values with the standard deviation ($\bar{T^{\prime }}\pm \sigma \left(T^{\prime }\right)$) of these sets of temperatures and the corresponding CVs were calculated when the radiance is simulated with an error of 3 times 0.01%. In particular, the target true temperature T and the Lambertian assumption with no emissivity error were also simulated. In addition, the average of the retrieved temperature in SWIR $\bar{T^{\prime }}$ was used as the target retrieved temperature. Its relative error with the true temperature is given by:

(19) $$\Delta T=\frac{\left|\bar{T^{\prime }}-T\right|}{T}$$

The obtained result (Table 2) shows that the system errors had a negligible effect on the dispersion degree of the temperatures retrieved from each of the SWIR bands. Therefore, the difference between the observed and simulated $\sigma \left(T^{\prime }\right)$ and CV for graphite and rock were mainly due to the Lambertian hypothesis.

#### 5.2.2. Errors from the Lambertian Assumption on Retrieval

It can be deduced from the previous section that the temperature inversion error caused by the systematic error was negligible. Therefore, by ignoring the error of the observation system, the retrieved temperature error caused by the emissivity was analyzed when it does not meet the Lambertian assumption. Assuming that the true temperature of the target is T, the target has directionality, and the true value of the emissivity is given by:

(20) $$\varepsilon \left(\theta_{r},\varphi_{r},\lambda \right)=1-R\left(\theta_{i},\varphi_{i};\theta_{r},\varphi_{r},\lambda \right)+\delta \varepsilon$$

where δε is the error term. 

According to Equation (15), the temperature inversion of each band in SWIR can then be computed as:

(21) $$T_{i}^{\prime }=\frac{hc}{k\lambda_{i}}ln^{-1}\left(\frac{2hc^{2}\left(\varepsilon_{i}+\delta \varepsilon_{i}\right)}{\lambda_{i}^{5}L_{i}}+1\right)=T{\left(1+\frac{\ln\left(\frac{2hc^{2}\delta \varepsilon_{i}}{\lambda_{i}^{5}L_{i}+2hc^{2}\varepsilon_{i}}+1\right)}{\ln\left(\frac{2hc^{2}\varepsilon_{i}}{\lambda_{i}^{5}L_{i}}+1\right)}\right)}^{-1},\left(i=0,\ldots ,n\right)$$

where $\lambda_{i}$ ranges between 1300 nm and 2500 nm and n is equal to 1200. 

It can be proved that, when $\delta \varepsilon_{i}=0$, $T_{i}^{\prime }=T$, when $\delta \varepsilon_{i}<0$, $T_{i}^{\prime }>T$, and when $\delta \varepsilon_{i}>0$, $T_{i}^{\prime }<T$. For the temperatures retrieved from all the SWIR bands, the total standard deviation $\sigma \left(T^{\prime }\right)$ and CV in *n* bands can be expressed as:

(22) $$\sigma \left(T^{\prime }\right)=\frac{1}{n-1}\sqrt{\sum_{i=0}^{n}{\left(T_{i}^{\prime }-\bar{T^{\prime }}\right)}^{2}},\left(i=0,\ldots ,n\right)\;$$

(23) $$\mathrm{CV}=\frac{\sigma \left(T^{\prime }\right)}{\bar{T^{\prime }}}=\frac{1}{n-1}\sqrt{\sum_{i=0}^{n}{\left(\frac{T_{i}^{\prime }}{\bar{T^{\prime }}}-1\right)}^{2}},\left(i=0,\ldots ,n\right)$$

where $\bar{T^{\prime }}$ is the retrieved temperature, and $\bar{T^{\prime }}$=$\frac{1}{n}\overset{n}{\underset{j=0}{\sum }}T_{j}^{\prime }$. Similarly, when $\delta \varepsilon_{i}=0$, $\sigma \left(T^{\prime }\right)=0$ and CV =0. When $\delta \varepsilon_{i}$ increases, $\sigma \left(T^{\prime }\right)$ and CV also increase. 

The computer simulation was used to further verify this analysis. By considering the retrieved temperature as the true temperature T, the emissivity errors were introduced using a computer simulation method, and the relative error and coefficient of variation were calculated. In particular, the emissivity error used two types of errors (Equi-Error (EE) and Stochastic Error (SE)) of the sinusoidal curve in the interval of 1–60%. EE simulates the same variation in error between the wavelengths. Therefore, the same emissivity error was added to each wavelength. The SE of the sinusoidal curve demonstrates that the errors between the wavelengths were different but had a certain correlation. Therefore, the stochastic error generated within the sinusoid was added to each wavelength.

**Figure 10:**
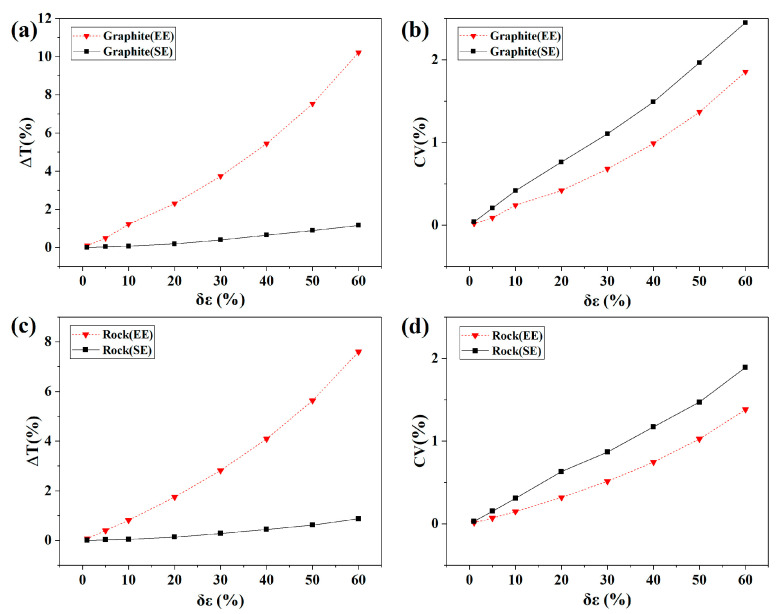
Plot of ΔT with the emissivity error for graphite (**a**) and rock (**c**), and plot of CVs with the emissivity error for graphite (**b**) and rock (**d**).

It can be seen from Figure 10 that, when the emissivity errors were 1%, 5%, and 10%, the temperature errors were in the range of 0.005–0.1%, 0.02–0.5%, and 0.04–1.22%, and the CVs were in the range of 0.014–0.04%, 0.07–0.25%, and 0.15–0.42%, respectively. ΔT and CV increased with the increase of δε, which confirms the conclusion of Equation (21). This also shows that the CV can reflect the error of emissivity and temperature. The CVs for the retrieved temperature of graphite and rock in this study were 0.17% and 0.24%, respectively. The emissivity errors of graphite and rock should be less than 5%, and the temperature inversion errors should be less than 0.5%. 

**Figure 11:**
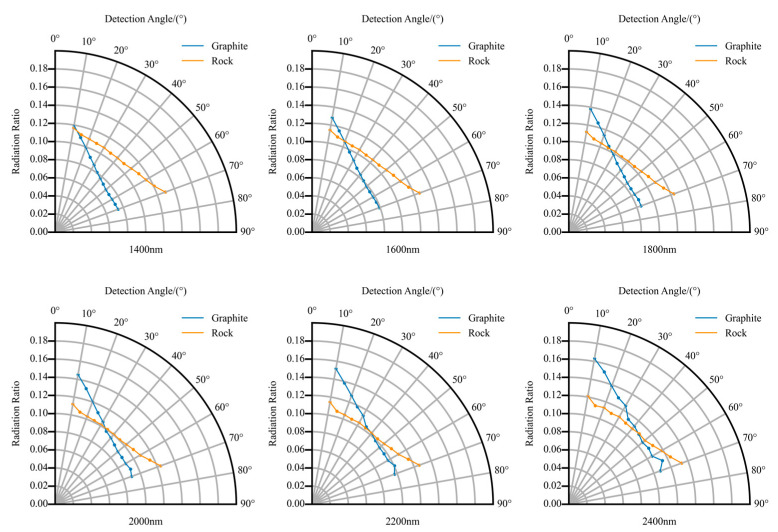
BRF distribution of the graphite and the rock in different wavelengths.

The BRF of the target was also tested through experiments. The observation direction was fixed as the normal direction, the incident angle of the light source was adjusted, and the BRF of the target was observed. The incident zenith angle of the light source changed from 10° to 70°. Figure 11a shows that the BRF of the graphite varied with an angle less than 0.05 ± 0.01, leading to an emissivity error less than 0.05 ± 0.01. Figure 11b shows that the BRF of the rock varied with an angle less than 0.02 ± 0.01. Therefore, the graphite has a slight directionality, and the rock is closer to Lambertian. Both can be regarded as Lambertian, and the errors of temperature inversion were all less than 0.5%.

When the errors of temperature inversion are less than 0.5% and the CVs of the temperatures are lower than 0.25%, the targets can be considered as Lambertian. The method can perform high-precision inversion for the target with Lambertian for negligible system error. The retrieval can be accurately performed as long as the relationship between the emissivity and BRF is clear.

## 6. Conclusions

This paper tackles the emissivity–temperature synchronous retrieval using a proposed multi-irradiance method without temperature measurement. Through additional irradiance conditions, the retrieval was first performed based on the principle of radiation transmission and energy conservation, using the correlation between the emission energy and the reflected energy of the high-temperature target, that is, the correlation between the emissivity and the BRF. An experimental device was then designed to perform remote sensing experimental observation of the target’s emission energy and reflected energy information. Finally, the experimental verification was conducted using the Lambertian graphite and rock materials. 

The normal spectral emissivity obtained of graphite and rock is consistent with related studies. In addition, the two sets of their temperatures that were retrieved from each of the 1201 SWIR bands (1300–2500 nm) had low dispersion with 763.24 ± 1.21 K and 581.62 ± 1.41 K, respectively. The retrieved temperature was slightly lower (by 2–3 K, 0.3–0.4%) than the inner temperature of samples measured by thermocouple. This demonstrates that the results of the spectral emissivity–temperature synchronous inversion are reasonable.

The retrieval errors originated from systematic errors, as well as errors in emissivity estimation due to the Lambertian assumption. The influence of the systematic error on temperature inversion is less than 0.05 K, which is negligible. After introducing the emissivity error term to simulate the non-Lambertian nature of the target, the relative errors and CVs of temperatures retrieved from all the SWIR bands increase with the increase of the emissivity error, in theory and simulation. When the errors of temperature inversion are less than 0.5% and the CVs of the temperatures are lower than 0.25%, the targets can be considered as Lambertian. It can be concluded that graphite and rock have a very slight non-Lambertian nature with emissivity errors less than 5%. The target temperatures are 763.24 K and 581.62 K, each with an error term less than 0.5%. This reveals that the multi-irradiance method can perform high-precision inversion for the Lambertian, and can also verify Lambertian nature by the dispersion degree of the temperatures retrieved of each SWIR band. 

It is expected that the proposed method can be applied to the simultaneous retrieval of the temperature and emissivity of high-temperature targets with stable temperature under the condition of the multi-irradiance of day and night. This study uses materials that are similar to Lambertian materials in order to demonstrate the experimental principles and methods. For the widely existing non-Lambertian materials, the relationship between the emissivity and the BRF, as well as the temperature error and correction, should be further studied. In addition, the validity of the method for a deeper temperature range and more kinds of materials from the experimental point of view should also be considered in future work.

## Figures and Tables

**Table 1:** Retrieved temperature in several wavelengths and measured temperatures.

Wavelength (nm)		1300	1500	1700	1900	2100	2300	2500	Measured Temperature (K)
Retrieved temperature (K)	Graphite	763.61	763.24	761.70	764.25	764.29	764.70	761.81	766.17 ± 0.62 K
	Rock	583.44	581.47	580.31	582.03	582.03	581.95	579.83	584.17 ± 0.59 K

**Table 2:** Statistics of temperatures retrieved in SWIR based on system error simulation.

	T (K)	$\bar{T^{\prime }}$ (K)	$\sigma \left(T^{\prime }\right)$ (K)	ΔT (%)	CV (%)
Graphite	582	582 ± 0.013	0.002	0.002	0.0004
Rock	763	763 ± 0.230	0.043	0.03	0.006
